# Cardiac function assessed by myocardial deformation in adult polycystic kidney disease patients

**DOI:** 10.1186/s12882-019-1500-1

**Published:** 2019-08-16

**Authors:** Mats C. H. Lassen, Atif N. Qasim, Tor Biering-Sørensen, Jacob L. T. Reeh, Terry Watnick, Stephen L. Seliger, Huanwen Chen, Mariem A. Sawan, Daniel Nguyen, Yongfang Li, Susie N. Hong, Meyeon Park

**Affiliations:** 1 Department of Cardiology, Herlev & Gentofte Hospital, University of Copenhagen, Niels Andersens vej 65, DK-2900, Post 835, Copenhagen, Denmark; 2 Division of Cardiology, University of California, San Francisco (UCSF), 505 Parnassus Ave, San Francisco, CA 94143 USA; 3 Division of Nephrology, University of Maryland School of Medicine, 655 W Baltimore S, Baltimore, MD 21201 USA; 4 Division of Nephrology, University of California, San Francisco (UCSF), 505 Parnassus Ave, San Francisco, CA 94143 USA; 5 Division of Cardiovascular Medicine, University of Maryland School of Medicine, 655 W Baltimore S, Baltimore, MD 21201 USA

**Keywords:** Autosomal dominant polycystic kidney disease, Two-dimensional speckle tracking echocardiography, Global longitudinal Strain

## Abstract

**Background:**

Patients with autosomal dominant polycystic kidney disease (ADPKD) have an increased risk of cardiovascular morbidity and mortality. Impaired left ventricular (LV) global longitudinal strain (GLS) can be a sign of subclinical cardiac dysfunction even in patients with otherwise preserved ejection fraction (EF). Transmitral early filling velocity to early diastolic strain rate (E/SRe) is a novel measure of LV filling pressure, which is often affected early in cardiac disease.

**Methods:**

A total of 110 ADPKD patients not on dialysis were included in this prospective study. All patients underwent an extensive echocardiographic examination including two-dimensional speckle tracking. GLS and strain rates were measured. The distribution of GLS and E/SRe was determined and patient characteristics were compared by median levels of GLS (− 17.8%) and E/SRe (91.4 cm). Twenty healthy participants were included as control group.

**Results:**

There was a significantly worse GLS in the ADPKD patients (mean: − 17.8 ± 2.5%) compared to the healthy controls (mean: − 21.9 ± 1.9%), *p* < 0.001. The same was true for E/SRe (mean: 10.0 ± 0.3 cm) compared to the control group (mean: 6.5 ± 0.3 cm), p < 0.001. In simple logistic regression, male gender (OR: 4.74 [2.10–10.71], *p* < 0.001), fasting glucose (odds ratio (OR) 1.05 [1.01–1.10], *p* = 0.024), htTKV (OR: 1.07 [1.01–1.13], *p* = 0.013), HDL cholesterol (OR: 0.97 [0.94, 0.996], *p* = 0.025), triglycerides (OR: 1.01 [1.00–1.02], *p* = 0.039), hemoglobin (OR: 1.50 [1.11–2.04], *p* = 0.009), and β-blocker use (OR: 1.07 [1.01, 1.13], *p* = 0.013) were all associated with higher GLS. After multivariate logistic regression with backward model selection, only male gender (OR: 5.78 [2.27–14.71], *p* < 0.001) and β-blocker use (OR: 14.00 [1.60, 122.51], *p* = 0.017) remained significant. In simple logistic regression models, BMI (OR: 1.11 [1.02–1.20], *p* = 0.015), systolic blood pressure (OR: 1.03 [1.00–1.06], *p* = 0.027) and β-blocker use (OR: 17.12 [2.15–136.20], *p* = 0.007) were associated with higher E/SRe - a novel measure of left ventricular filling pressure. After backward elimination, only β-blocker use (OR: 17.22 [2.16, 137.14], *p* = 0.007) remained significant.

**Conclusion:**

Higher GLS and E/SRe are common in ADPKD patients, even in patients with preserved eGFR and normal left ventricular EF. GLS and E/SRe may aid in cardiovascular risk stratification in patients with ADPKD as they represent early markers of cardiac dysfunction.

**Electronic supplementary material:**

The online version of this article (10.1186/s12882-019-1500-1) contains supplementary material, which is available to authorized users.

## Introduction

Individuals with autosomal dominant polycystic kidney disease (ADPKD) have a high prevalence of cardiovascular (CV) disease associated with significant morbidity and mortality [1]. Death due to cardiovascular disease has been reported to be as high as 33% in ADPKD patients with end-stage renal disease, primarily due to ischemic heart disease and congestive heart failure [1].

Left ventricular hypertrophy is a strong independent risk factor for CV morbidity and mortality and is highly prevalent in ADPKD patients, even in the absence of hypertension [2, 3]. Although results from more recent studies suggest a lower occurrence of cardiac remodeling in ADPKD patients, possibly due to earlier antihypertensive treatment [4], structural and functional abnormalities remain common in these patients [5]. Furthermore, both hypertensive and normotensive ADPKD patients show significant diastolic dysfunction, suggesting cardiac pathology early in ADPKD [5]. Although these abnormalities become more severe at advanced stages of chronic kidney disease (CKD), they can be detected in individuals with preserved eGFR [6].

**Fig. 1:**
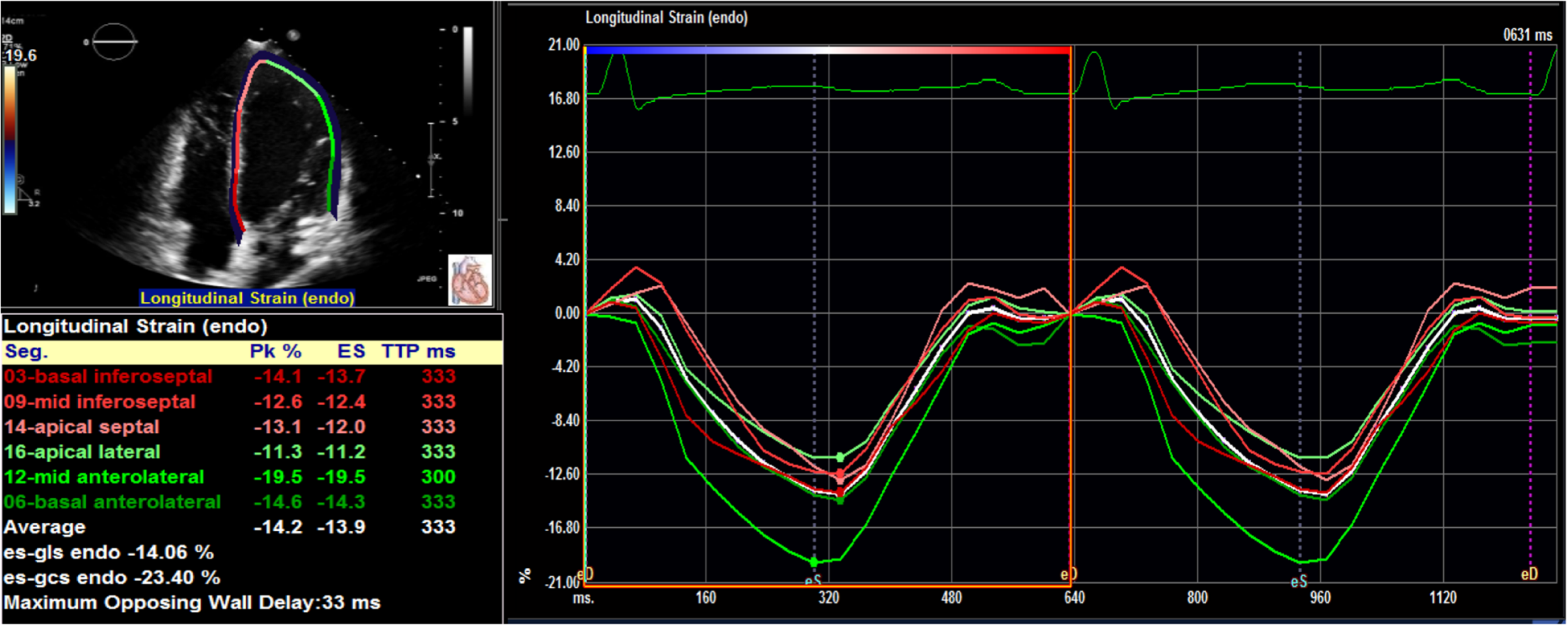
Example of GLS measurement. Abbreviations: GLS = global longitudinal strain

Previous understanding of CV risk in ADPKD has been achieved primarily through measurements of echocardiograms and cardiac magnetic resonance imaging [3, 4]. The recent results of the HALT-PKD study demonstrated that early treatment with angiotensin-converting enzyme inhibitors (ACEI) to a goal systolic blood pressure < 110 mmHg was associated with a greater decline in left ventricular mass index among individuals aged 15–49 with early-stage ADPKD compared to a standard blood pressure target [7]. These results may encourage earlier diagnosis and treatment of family members at risk for ADPKD. More sensitive echocardiographic techniques well-correlated to CV risk are needed in this younger asymptomatic group. Two-dimensional speckle-tracking is a technique that assesses regional deformation along infinitesimally small axes [8]. Global longitudinal strain (GLS), assessed by two-dimensional speckle tracking, has emerged as a new measure of left ventricular (LV) dysfunction [9]. GLS is a direct non-invasive quantitative measure of the myocardial contractility of the left ventricle. The lower (i.e. more negative) GLS is, the better contractility (Fig. 1). GLS can be altered despite preserved LV ejection fraction (LVEF) in various conditions predisposing to cardiovascular morbidity, including pre-diabetics with normal blood pressure [10], diabetes [11] and hypertension [12]. A peak GLS in the range of − 20% ± 2% can be expected in a healthy person [13].

In a population of asymptomatic dialysis patients, two-dimensional speckle-tracking showed significantly higher (less negative) values of left ventricular peak longitudinal strain and early diastolic strain when compared to healthy individuals [14]. Speckle tracking echocardiography has been used to detect and evaluate myocardial ischemia, valvular heart disease, dyssynchrony, arrhythmias, cardiomyopathies and assessment of diastolic function [8]. Furthermore, speckle tracking has been demonstrated to detect myocardial fibrosis in more advanced stages of kidney disease [15], and is an important feature of uremic cardiomyopathy in these patients.

Two-dimensional speckle-tracking has not previously been used to assess cardiac function in individuals with ADPKD. The aim of our study was 1) to investigate the distribution of GLS as assessed by two-dimensional speckle-tracking in asymptomatic patients with ADPKD and 2) to identify clinical variables associated with higher (less negative) strain measurements in these patients.

## Methods

### Study population

We performed a cross-sectional study in the Baltimore PKD Center Core ADPKD cohort. Patients ≥18 years old with ADPKD as defined by the modified Pei-Ravine criteria [16] and with eGFR> 15 ml/min/1.73m^2^ were eligible to participate in a prospective observational cohort study at the Baltimore PKD Center at the University of Maryland School of Medicine. Patients with prior kidney transplant, pregnancy, uncontrolled diabetes (defined as a glycated hemoglobin > 7% or use of more than one anti-diabetic medication), current participation in an interventional pharmaceutical clinical trial, any systemic disease (e.g., lupus) likely to lead to kidney disease, or suspected kidney disease other than ADPKD were excluded. Participants were enrolled from 2013 through 2017. Family and personal medical history including absence of cardiac disease was obtained by nephrologist investigators through patient interview and review of medical records. Serum glucose, lipids and creatinine (using an IDMS-traceable assay) were measured after an overnight fast, and GFR was estimated using the creatinine-based CKD-Epi estimating equation [17]. Albuminuria was estimated as the ratio of urinary albumin to creatinine in a spot morning sample. Blood pressure was measured in the seated position and the average of 3 measures was calculated. Abdominal MRI was performed, and total kidney volume was calculated by a single radiologist blinded to the clinical characteristics and cardiac measurements of participants. TKV was indexed to height (htTKV, expressed in cc/meters). Echocardiograms were performed on all participants at baseline. Of the 126 patients enrolled in this cohort, *N* = 16 were excluded due to missing echocardiogram images or inadequate image quality for speckle tracking analysis, leaving 110 patients included in this study.

A control group consisting of 20 healthy age and gender matched controls were included as a comparison. All controls underwent an extensive echocardiographic examination and personal medical history was obtained. Healthy controls did not undergo abdominal MRI, therefore TKV could not be measured for the controls.

### Echocardiography

Experienced clinicians and sonographers performed all echocardiographic examinations under a standard echocardiography protocol using 2-dimensional-, spectral- and tissue Doppler. All images were acquired on Philips cardiology ultrasound systems (EPIC7, iE33), stored as DICOM images on the Synapse Cardiovascular platform (Fuji Medical, version 4.0.8 SR1) at the University of Maryland Medical Center, and analyzed offline at UCSF using vendor-independent post-processing software TomTec-Arena 2D-CPA by a trained investigator.

Conventional two-dimensional echocardiography: Left ventricle dimensions (interventricular septal thickness (IVSd), LV internal diameter (LVIDd) and LV posterior wall thickness (PWTd)) were measured on the parasternal long-axis projection. This was done in end-diastole at the tip of the mitral valve leaflets as per ASE guidelines [13]. LV mass was calculated using area length method using 2D and then divided by body surface area to get LV mass index (LVMI). LA diameter was measured in the parasternal long-axis view by using the leading edge to leading edge method. LV ejection fraction (LVEF) was measured using the modified Simpson’s biplane method in the apical 4- and 2-chamber projections.

Pulsed-wave (PW) Doppler imaging was used in the 4-chamber view to obtain peak early filling (E-wave), atrial filling (a-wave) and deceleration time (DT). PW tissue Doppler imaging was used to obtain early (e’) peak myocardial diastolic velocity at the lateral and septal mitral annular segments. The mean was calculated and used for determining E/e’.

Speckle tracking was performed in the apical 4-, 2- and 3-chamber projections as recommended by current guidelines [13]. The tracing of the myocardial wall was done using a semiautomatic function. In cases of inaccurate tracing, the investigator would manually adjust the tracing. A total of 18 segments, 6 from each projection, were included. A total of 7 subjects had a maximum of 2 segments excluded in the cohort. All 3 apical projections were available for speckle tracking in all but 1 patient. GLS was calculated as the mean peak strain of the three apical projections. E/e’sr was calculated as E velocity (m/s) divided by the absolute value of global peak early diastolic strain rate (e’sr) averaged across 3 apical views. The investigator could exclude segments, if considered untraceable. Total study population was stratified according to the median GLS (− 17.8%) to identify clinical factors associated with higher (less negative) GLS. Our lab has previously reported good intra- and interobserver variability of both GLS and SRe with a small bias (GLS: mean difference ± 1.96 SDs was 0.1 ± 1.6% for the intraobserver analysis and − 0.8 ± 2.0% for the interobserver analysis) and (SRe: − 0.06 ± 0.25 for the intraobserver analysis and 0.06 ± 0.28 for the interobserver analysis) [18]. Diastolic function was assessed according to existing recommendations [19].

### Statistical methods

We divided the cohort into groups with GLS above and below the median value (− 17.8%). Baseline demographic, clinical and echocardiographic data were compared between those with higher (below median) GLS and lower (above median) GLS. Kruskal-Wallis test was used for comparing non-Gaussian distributed continuous variables – estimated GFR, micro albumin/creatinine ratio, triglycerides, htTKV, left ventricle mass index, and E/e’ ratio. Student’s T-test was used for comparing continuous variables between groups, and Chi-squared test was used for comparing categorical variables. Student’s T-test was also used for comparing the control group to the ADPKD group. All covariates were tested for statistical differences between those with higher and lower GLS (stratified according to the population median of GLS (− 17.8%)) as has previously been done [20]. Correlations between eGFR and htTKV with GLS were defined using Pearson correlation coefficient. Covariates with a *p*-value < 0.15 in simple regression analyses were included in multiple linear regression with stepwise backward elimination. The threshold to remove was 0.05. Logistic regression was used to calculate odds ratios for the predictors of higher (worse) GLS and higher (worse) E/early diastolic strain rate, defined by ≤91.4 cm. Simple and multiple logistic regression models with backward elimination were built. Data analyses were performed using SAS 9.4 (SAS Institute, NC).

## Results

### Baseline characteristics

**Fig. 2:**
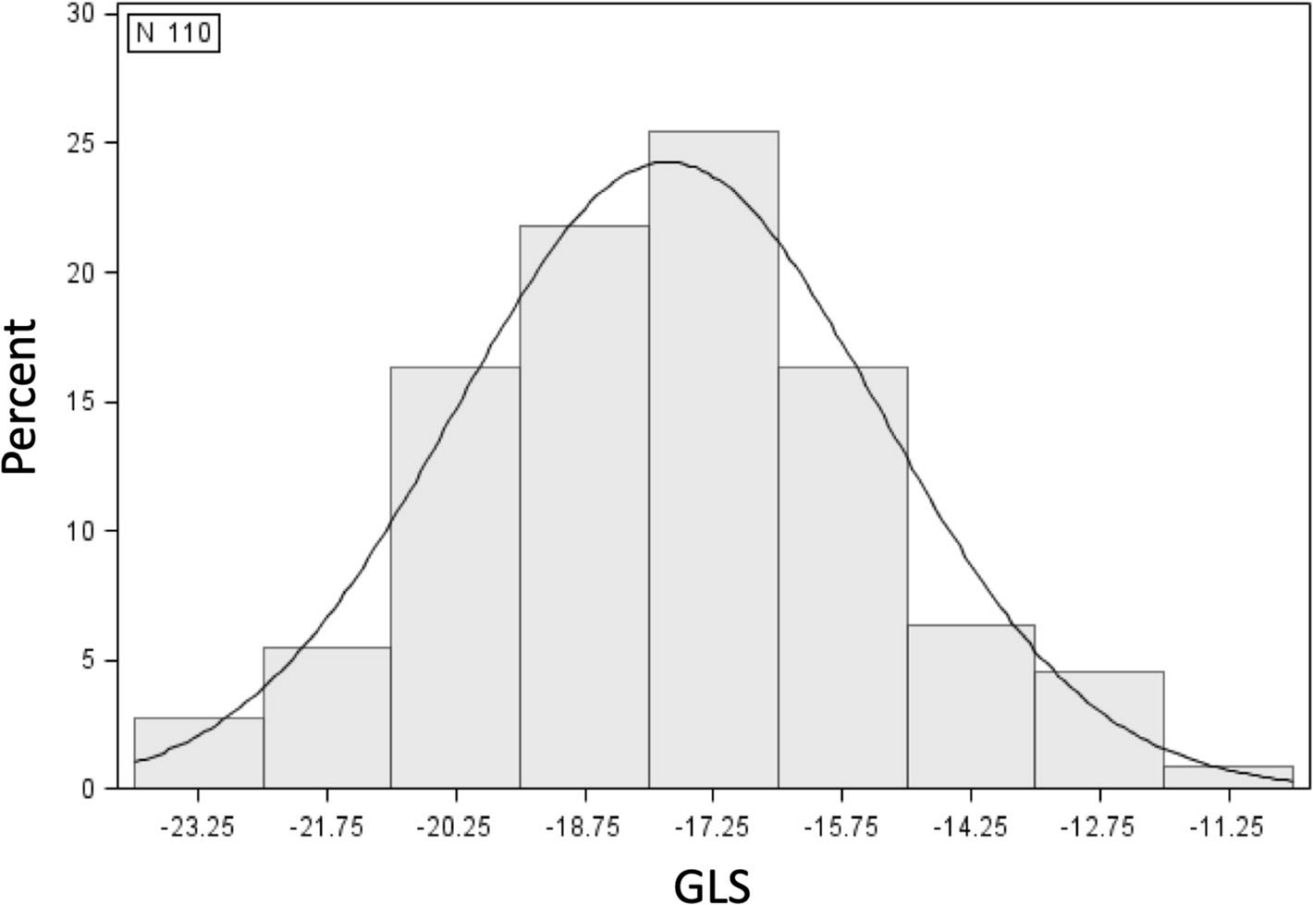
Distribution of GLS in the study population. Abbreviations: GLS = global longitudinal strain

**Fig. 3:**
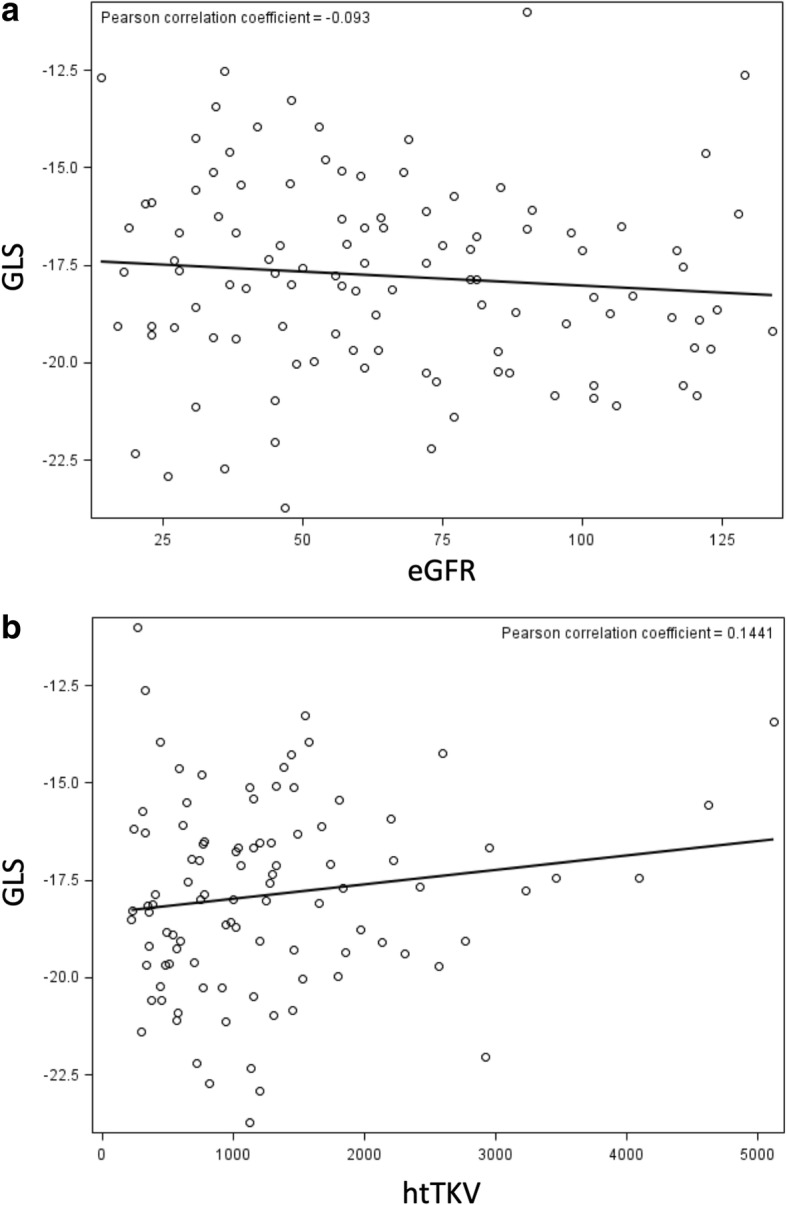
**a** Scatterplot showing the correlation between eGFR and GLS. Abbreviations: GLS = global longitudinal strain, eGFR = estimated glomerular filtration rate. **b** Caption: Scatterplot showing correlation between htTKV values and GLS. Abbreviations: GLS = global longitudinal strain, htTKV = height-indexed total kidney volume

Our cohort comprised 110 ADPKD patients (mean ± SD age 46.1 ± 13.3 years). Forty-eight (44%) were men and 92 (84%) were Caucasian. Clinical characteristics of the study population are displayed in Table 1. The mean ± SD of GLS was − 17.8 ± 2.5% (Fig. 2). GLS correlated weakly with eGFR (Pearson correlation coefficient = − 0.093) (Fig. 3a) and more strongly with htTKV (Pearson correlation coefficient = 0.144) (Fig. 3b). Patients with higher (worse) GLS were more likely to be male, have higher levels of fasting glucose and hemoglobin, be on ß-blockers and have lower levels of high-density lipoprotein despite no statistically significant differences in age, race, blood pressure and micro albumin/creatinine ratio (Table 1).

**Table 1:** Baseline characteristics

	Lower (better) strain GLS = <− 17.8% (*n* = 55)	Higher (worse) strain (GLS > -17.8%) (n = 55)	*P Value*
Demographics			
Age, years	45.1 ± 12.4	47.2 ± 14.3	0.43
Male gender, n (%)	14 (25)	34 (62)	< 0.001
Race, n (%)			
Caucasian	48 (87)	44 (80)	0.61
African American	5 (9)	8 (15)	
Asian	2 (4)	3 (5)	
Clinical data			
Systolic Blood Pressure, mmHg	124.3 ± 13.4	127.6 ± 15.3	0.24
Diastolic Blood Pressure, mmHg	76.0 ± 10.3	79.4 ± 9.7	0.08
Estimated GFR, mL/min/1.73m^2^	66 [45–102]	57 [36–80]	0.12
Microalbumin/creatinine ratio, mg/g	20 [10–30]	21 [12–59]	0.27
Ht-Indexed Total Kidney Vol, cm^3^/m	819 [480–1304]	1289 [738–1806]	0.01
BMI, kg/m^2^	27.3 ± 5.7	28.8 ± 4.7	0.13
Fasting Glucose, mg/dL	83.6 ± 9.0	87.8 ± 9.5	0.02
HDL cholesterol, mg/dL	53.0 ± 14.5	47.0 ± 12.2	0.02
LDL cholesterol, mg/dL	105.5 ± 33.3	106.2 ± 30.5	0.91
Total Cholesterol, mg/dL	178.5 ± 33.9	179.2 ± 40.4	0.92
Triglycerides, mg/dL	86 [64–112]	96 [75–126]	0.13
Hemoglobin, g/dL	12.5 ± 1.3	13.2 ± 1.3	0.01
ACE Inhibitor, n (%)	37 (69)	37 (67)	0.89
Beta blocker, n (%)	1 (2)	13 (24)	0.001
Diuretic, n (%)	5 (9)	4 (7)	0.74

### Comparison of control group to ADPKD group

There was a higher GLS in the ADPKD patients (mean: − 17.8 ± 2.5, 95%CI [− 18.3-(− 17.4)]) compared to the healthy controls (mean: − 22.0 ± 2.2, 95% CI [− 23.0-(− 21.0)]) (*p* < 0.001). E/SRe was increased (worse) in the ADPKD patients (mean: 100.3 ± 31.5 cm, 95% CI [94.4–100.6]) compared to the control group (mean: 65.2 ± 15.0 cm, 95% CI [58.2–72.3]) (*p* < 0.001). (Table 2).

**Table 2:** Control group and ADPKD group

	ADPKD group (*n* = 110)	Control group (*n* = 20)	*P Value*
Demographics			
Age, years	46.1 ± 13.3	46.1 ± 11.0	0.99
Male gender, n (%)	48 (43.6)	8 (40.0)	0.76
Race, n (%)			
Caucasian	92 (83.6)	17 (85.0)	0.43
African American	13 (11.8)	1 (5.0)	
Asian	5 (4.5)	2 (10.0)	
Clinical data			
Systolic Blood Pressure, mmHg	126 ± 14	122 ± 14	0.26
Diastolic Blood Pressure, mmHg	78 ± 10	71 ± 9	0.004
Estimated GFR, mL/min/1.73m^2^	60.7 [38.0–88.0]	99.5 [76.5–113.5]	< 0.001
BMI, kg/m^2^	28.5 ± 6.6	26.6 ± 3.2	0.21
HDL cholesterol, mg/dL	50.0 ± 13.7	55.6 ± 15.0	0.10
LDL cholesterol, mg/dL	105.8 ± 31.8	120.4 ± 34.8	0.07
Total Cholesterol, mg/dL	178.8 ± 37.1	194.1 ± 37.6	0.09
Triglycerides, mg/dL	94 [70–121]	84 [58–108]	0.29
Hemoglobin, g/dL	12.9 ± 1.3	13.9 ± 1.5	0.005
ACE Inhibitor, n (%)	74 (67.9)	1 (5.0)	< 0.001
Beta blocker, n (%)	14 (12.8)	0 (0)	0.09
Echocardiographic data			
Left Ventricle Ejection Fraction, %	59.8 ± 3.6	66.1 ± 3.3	< 0.001
E peak, cm/s	71.0 ± 16.1	77.2 ± 11.6	0.11
E/e’ ratio	7.2 [6.3–9.1]	6.7 [6.1–7.6]	0.17
Global Longitudinal Strain, %	−17.9 ± 2.5	−22.0 ± 2.2	< 0.001
Systolic Strain Rate, s^− 1^	0.78 ± 0.13	1.05 ± 0.11	< 0.001
Early Diastolic Strain Rate, s^1^	0.75 ± 0.19	1.23 ± 0.30	< 0.001
E/early diastolic strain rate, cm	100.3 ± 31.5	65.2 ± 15.0	< 0.001
IVSd, cm	0.96 ± 0.15	0.91 ± 0.16	0.28
LVIDd, cm	4.8 ± 0.5	4.4 ± 0.5	0.01
LVPWd, cm	0.94 ± 0.14	0.91 ± 0.16	0.36
Left ventricular mass index, (g/m^2^)	82.0 ± 21.6	69.1 ± 8.5	0.014
Left atrial volume index, (mL/m^2^)	21.3 ± 2.8	23.4 ± 5.1	0.013

*IVSd* Interventricular septal thickness, *LVIDd* Left ventricular internal diameter, *LVPWd* Left ventricular posterior wall, *E* Peak early filling velocity

### Conventional and 2D speckle tracking results

Comparisons of echocardiographic parameters between those with higher and lower GLS are described in Table 3. On conventional echocardiogram, most measurements were not significantly different between groups of higher versus lower GLS. The difference in LVEF was statistically significant but was within the range of normal (60 versus 59%) for both groups. Similarly, although those with higher GLS had thicker interventricular septal diameter (IVSd) and thicker left ventricle posterior wall (PWTd) that reached statistical significance, the difference in these measures (0.04 cm, or 0.4 mm) was below the limit of detection by standard techniques (1 mm). On 2D speckle tracking, in the group with higher (less negative / worse) GLS, subjects had lower systolic and diastolic strain rates, increased E/SRe (a load-independent marker of LV filling pressure).

**Table 3:** Echocardiographic data

	Lower (better) strain GLS = <− 17.8% (n = 55)	Higher (worse) strain (GLS > -17.8%) (n = 55)	*P Value*
Conventional Echo			
Left Ventricle Ejection Fraction, %	60.6 ± 3.8	59.0 ± 3.3	0.02
Left Ventricle Mass index, g/m^2^	80 [66–91]	82 [71–95]	0.34
E peak, cm/s	74.9 ± 17.1	67.2 ± 14.2	0.01
E/e’ ratio	7.33 [6.1–9.5]	7.20 [6.3–8.6]	0.60
Diastolic function, n (%)			
Normal Diastolic Function	53 (96)	48 (87)	0.16
Indeterminate Diastolic Function	2 (4)	7 (13)	
Diastolic Dysfunction	0 (0)	0 (0)	
Speckle Echo			
Global Longitudinal Strain, %	−19.8 ± 1.4	−15.8 ± 1.6	< 0.001
Systolic Strain Rate, s^− 1^	0.9 ± 0.1	0.7 ± 0.1	< 0.001
Early Diastolic Strain Rate, s^1^	0.9 ± 0.2	0.6 ± 0.2	< 0.001
E/early diastolic strain rate, cm	87.9 ± 20.3	111.3 ± 36.6	< 0.001
IVSd, cm	0.9 ± 0.1	1.0 ± 0.2	< 0.001
LVIDd, cm	4.7 ± 0.5	4.8 ± 0.5	0.21
PWTd, cm	0.9 ± 0.1	1.0 ± 0.1	0.002
LAVI, mL/m^2^	21.8 ± 2.6	20.9 ± 3.0	0.08

*IVSd* Interventricular septal thickness, *LVIDd* Left ventricular internal diameter, *LVPWd* Left ventricular posterior wall, *E* Peak early filling velocity

### Covariate associations with GLS and E/SRe

In simple linear regression models, male gender, diastolic blood pressure, HDL, triglycerides, hemoglobin, β-blocker use were associated with higher GLS. After backwards elimination, age, male gender, triglycerides, diastolic blood pressure, eGFR and β-blocker use were independently associated with GLS (Table 4). In simple linear regression models, factors associated with greater E/SRe were systolic blood pressure, micro albumin/creatinine ratio, and β-blocker use. In the multiple model, systolic blood pressure and β-blocker use were associated with higher E/SRe (Table 5).

**Table 4:** Associations with GLS, Linear Regression

Variable	Univariate		Multiple linear regression with backward model selection	
	Beta coefficient (95% CI)	*P* value	Beta coefficient (95% CI)	*P* value
Age	0.017 (−0.018,0.052)	0.34	0.053 (0.007,0.10)	0.026
Gender	1.61 (0.73,2.51)	< 0.001	1.58 (0.70,2.46)	< 0.001
Diastolic Blood Pressure, mmHg	0.055 (0.01,0.10)	0.017	0.06 (0.015,0.10)	0.001
Estimated GFR, per 10 mL/min/1.73m^2^	−0.072 (− 0.22,0.076)	0.33	0.23 (0.044,0.42)	0.016
Ht-Indexed Total Kidney Vol, per 100 cm^3^/m	0.037 (−0.015,0.089)	0.16		
BMI, kg/m^2^	0.08 (−0.008,0.17)	0.075		
Fasting Glucose, mg/dL	0.039 (−0.01,0.088)	0.12		
HDL cholesterol, mg/dL	−0.047 (− 0.08,-0.014)	0.006		
Triglycerides, mg/dL	0.007 (0,0.014)	0.04	0.007 (0.001,0.013)	0.034
Hemoglobin, g/dL	0.44 (0.10,0.76)	0.012		
Beta blocker	2.73 (1.42,4.04)	<.0001	2.26 (0.86,3.65)	0.002

**Table 5:** Associations with E/SRe, Linear Regression

Variable	Univariate		Multiple linear regression with backward model selection	
	Beta coefficient (95% CI)	P value	Beta coefficient (95% CI)	P value
Age	0.35 (−0.01, 0.80)	0.12		
Systolic Blood Pressure, mmHg	0.51 (0.10, 0.92)	0.014	0.43 (0.03, 0.83)	0.04
Estimated GFR, per 10 mL/min/1.73m^2^	−0.67 (−2.57, 1.23)	0.48		
BMI, kg/m2	1.11 (−0.023, 2.25)	0.06		
Microalbumin/creatinine ratio, mg/g	0.06 (0.006, 0.12)	0.03		
ACE Inhibitor	−11.9 (−24.6, 0.9)	0.07		
Beta blocker	29.6 (12.4, 46.8)	< 0.001	27.4 (10.2, 44.6)	0.002

### Logistic regression with GLS and E/diastolic strain rate

In simple logistic regression, male gender (OR: 4.74 [2.10, 10.71], *p* < 0.001), htTKV (OR: 1.07 [1.01–1.13], *p* = 0.013), fasting glucose (OR: 1.05 [1.01–1.10], *p* = 0.024), HDL (OR: 0.97 [0.94, 0.996], *p* = 0.025), hemoglobin (OR: 1.50 [1.11–2.04], *p* = 0.009), triglycerides (OR: 1.01 [1.00–1.02], *p* = 0.039), and β-blocker use (OR: 16.40 [2.06, 13.47], *P* = 0.008) were all associated with higher GLS (Table 6). htTKV was associated with GLS even after adjusting for eGFR (OR: 1.08 [1.02–1.15], *p* = 0.014). According to the results of multiple logistic regression with backward elimination, male gender (OR: 5.78 [2.27–14.71], *p* < 0.001) and β-blocker use (OR: 14 [1.60–122.51], *p* = 0.017) were found to be strongly associated with higher GLS.

**Table 6:** Associations with GLS: Logistic regression

Variable	Univariate		Multiple logistic regression with backward model selection	
	Odds ratio (95% CI)	*P* value	Odds ratio (95% CI)	*P* value
Age	1.01 (0.98, 1.04)	0.42		
Gender	4.7 (2.1, 10.7)	< 0.001	5.8 (2.3 14.7)	< 0.001
Diastolic Blood Pressure, mmHg	1.03 (1.0, 1.1)	0.09		
Estimated GFR, per 10 mL/min/1.73m^2^	0.90 (0.80, 1.02)	0.10		
BMI, kg/m^2^	1.06 (0.98, 1.14)	0.13		
Fasting Glucose, mg/dL	1.05 (1.01, 1.10)	0.02		
HDL cholesterol, mg/dL	0.97 (0.94, 0.99)	0.03		
Triglycerides, mg/dL	1.01 (1.0, 1.02)	0.04		
Hemoglobin, g/dL	1.50 (1.1, 2.04)	0.01		
Beta blocker	16.4 (2.1, 130.5)	0.01	14 (1.6, 122.5)	0.017
Ht-Indexed Total Kidney Vol, per 100 cm^3^/m	1.07 (1.01, 1.13)	0.01		

The same method was used to investigate predictors of elevated filling pressure as assessed by E/SRe. Simple logistic regression showed that BMI (OR: 1.11 [1.02–1.20], *p* = 0.015), systolic blood pressure (OR: 1.03 [1.00–1.06], *p* = 0.027) and β-blocker use (OR: 17.12 [2.15–136.2], *p* = 0.007) were all independently associated with an increased E/SRe (> 91.4 cm). Based on the results of multiple variables logistic regression with stepwise backward elimination, only one variable remained significant: β-blocker use (OR: 17.22 [2.16–137.14], *p* = 0.007 (Table 7).

**Table 7:** Associations with E/SRe: Logistic regression

Variable	Univariate		Multiple logistic regression with backward model selection	
	Odds ratio (95% CI)	*P* value	Odds ratio (95% CI)	*P* value
Age	1.02 (0.99, 1.05)	0.11		
Systolic Blood Pressure, mmHg	1.03 (1.0, 1.06)	0.03		
Estimated GFR, per 10 mL/min/1.73m^2^	0.91 (0.81, 1.03)	0.13		
BMI, kg/m2	1.11 (1.02, 1.20)	0.02		
Microalbumin/creatinine ratio, mg/g	1.01 (0.99, 1.01)	0.09		
ACE Inhibitor	0.54 (0.24, 1.22)	0.14		
Beta blocker	17.1 (2.2, 136.2)	0.01	17.2 (2.2, 137.1)	0.007

### Sensitivity analysis

Because the number of individuals on beta blockers in this cohort was quite low, and because patients on beta blockers may represent a sub-group with more advanced cardiac dysfunction, we performed sensitivity analyses excluding individuals on beta blockers. In these analyses, the major findings in adjusted linear regression showed that age, gender, diastolic blood pressure, and estimated GFR were still significantly associated with higher GLS (Additional file 1 Table S1A), and systolic blood pressure and ACE inhibitor use remained significant predictors of E/SRe (Additional file 1 Table S1B). The major findings in logistic regression models with backward elimination showed that gender was significantly associated with higher GLS (Additional file 1 Table S2A), and BMI remained significantly associated with E/SRe (Additional file 1 Table S2B).

## Discussion

In this prospective cohort study of adult ADPKD patients with no history of cardiac disease, we described the distribution of GLS relative to established clinical predictors of disease severity in ADPKD. Given that GLS has been demonstrated as an early and sensitive predictor of cardiovascular morbidity and mortality in other patient populations [21–23], it is notable that the ADPKD patients in this study with otherwise normal conventional echocardiographic examinations displayed a relatively high GLS (mean: − 17.8% ± 2.5%) compared to the healthy age and gender matched control group (*mean: − 21.9 ± 1.9%).* The values found in our control group were similar to normative values in a large meta-analysis in healthy individuals (mean: − 19.7% +/− 0.4%) [24]. As both LVEF and GLS are measures of systolic function, this finding underscores the role of GLS as an early and sensitive marker of cardiac pathology. As patients may be encouraged to seek diagnosis earlier in light of recent findings of potential benefits of more aggressive blood pressure control as per HALT-PKD, GLS may represent a method by which to risk-stratify asymptomatic patients at risk for subclinical cardiovascular disease and adverse cardiovascular outcomes. Our findings of early subclinical dysfunction highlight the need to focus on early interventions to improve cardiovascular outcomes in ADPKD patients.

Longitudinal strain is known to be influenced by several clinical factors such as diabetes [11], obesity [25] and hypertension [12] and generally declines in cardiac pathologic states before there is a noticeable reduction in LVEF. This may be due to the superior sensitivity of the strain technique when compared to conventional echocardiographic measures. Although LVEF and GLS are physiologically related, as they are both measures of systolic function, they reflect different aspects of the myocardial deformation thus explaining the weak correlation observed in this study. LVEF measures a change in volume and predominantly quantifies radial contraction, whereas GLS represents contraction of the longitudinal myocardial fibers. Sub-endocardial longitudinal fibers are sensitive to ischemia, and both fibrosis and lipid deposition have been shown to affect GLS [26, 27]. In mouse models of PKD, strain imaging has also demonstrated myocardial dysfunction, which is rescued by suppression of galectin-3, a protein regulator of fibrosis that may aggravate the cystic phenotype of autosomal recessive PKD [28].

As more cysts develop, the renin-angiotensin-aldosterone system (RAAS) is activated leading to high blood pressure very early in the pathogenesis [29]. Therefore, greater RAAS activation may explain the association of greater htTKV with lower GLS independent of blood pressure effects. A significant association was also seen between blood pressure and GLS. This association may be due to the fact that longitudinal strain is affected by hypertrophic LV remodeling and afterload conditions. This is especially true in patients suffering from ADPKD as these individuals are more prone to undergo hypertrophic LV remodeling due to uremic cardiomyopathy, early-onset hypertension, and hyperinsulinemia [3, 30, 15]. GLS may thus be able to detect even subtle increases in afterload in these otherwise asymptomatic individuals. However, as kidney disease in general affects cardiac function, it is difficult to conclude whether specific ADPKD-pathogenic mechanisms are responsible for the findings or whether kidney dysfunction alone was the cause. The association between htTKV and lower GLS independent of eGFR could indicate that ADPKD-specific mechanisms play a role, but it is likely a combination of both factors. The fact that hemoglobin was higher in the group with higher GLS could be due to the increased EPO concentration sometimes observed in more advanced stages of ADPKD leading to a higher level of hemoglobin [31, 32]. However this is mostly speculative and future studies are needed to elucidate how EPO concentrations and hemoglobin levels in ADPKD patients affects echocardiographic parameters.

Diastolic dysfunction assessed according to current recommendations [19] was present in only a small minority of participants. This was interesting, as elevated LV filling pressure assessed by E/SRe was found to be quite high in our population. This was especially true in the group with worse GLS (> − 17.8%), where the mean of E/SRe (111.29 ± 36.57) were similar to the ranges of this measure reported in studies of STEMI patients [33], heart failure patients [34] and even higher than in atrial fibrillation patients [35]. This has been suggested as a more sensitive non-invasive measure of LV filling pressure. In our population of ADPKD patients, a significant number appear to have elevated LV filling pressures when assessed by E/SRe, despite having relatively normal blood pressure, LVMI and preserved ejection fraction. We found BMI, systolic blood pressure and β-blocker use to be independent predictors of increased E/SRe. That BMI and systolic blood pressure were associated with elevated filling pressure may be due to the adverse effect on LV relaxation which co-morbid conditions such as hypertension and metabolic syndrome can have [26, 36]. The fact that β-blocker use was an independent predictor of both E/SRe and GLS is possibly due to reverse causality in which the group of patients using β-blockers may have more severe hypertension and/or more pronounced impaired cardiac function than the larger group of patients not using β-blockers.

We also found that more men had higher GLS than women. This is consistent with reports that CV disease in men with ADPKD is more severe [3] and it is well known that women have higher LVEF and lower GLS than men [21]. The lack of an association between albuminuria and GLS was surprising, and may represent a low prevalence of albuminuria in our cohort.

Our study had some limitations. This was a single-center study. Participants had relatively preserved renal function, and the majority were Caucasian. Although none of these patients had diabetes requiring insulin, we do not have data on measures of insulin sensitivity. Furthermore, we did not have information regarding treatment with erythropoietin stimulating agents (ESA). Nevertheless, this is the first study to our knowledge to apply speckle echo images in adults with ADPKD. Although speckle echo represents a novel technology not generally available in general clinical practice, strain measurements can be applied to standard echo images, making this approach potentially easier and more cost effective for clinical risk stratification than cardiac MRI [4]. Finally, although we adjusted for eGFR, we cannot account for residual confounding including by kidney dysfunction independent of ADPKD.

## Conclusion

Higher (worse) GLS and elevated LV filling pressure assessed by E/SRe are common in patients suffering from autosomal dominant polycystic kidney disease, even those with preserved eGFR and LVEF. These measures may represent early markers of cardiac dysfunction that may be used for cardiovascular risk stratification in autosomal dominant polycystic kidney disease. Use of sensitive measurements from speckle echo may be of benefit in elucidating mechanisms of early cardiovascular disease onset in early stage ADPKD.

## Additional file

Additional file 1: **Table S1A.** Associations with GLS, Linear Regression (those who were on Beta blocker were excluded from analysis). **Table S1B.** Associations with E/SRe, Linear Regression (those who were on Beta blocker were excluded from analysis). **Table S2A.** Associations with GLS: Logistic regression (those who were on Beta blocker were excluded from analysis). **Table S2B.** Associations with E/SRe: Logistic regression (those who were on Beta blocker were excluded from analysis). (DOCX 18 kb)

## Data Availability

The datasets used and/or analyzed during the current study are available from the corresponding author on reasonable request.

## Abbreviations

ADPKD: Autosomal dominant polycystic kidney disease
BMI: Body mass index
CKD: Chronic kidney disease
CV: Cardiovascular
E/SRe: Ratio of transmitral early filling velocity to early diastolic strain rate
eGFR: Estimated glomerular filtration rate
GLS: Global longitudinal strain
HDL: High density lipoprotein
htTKV: Height indexed total kidney volume
IVSd: Interventricular septal thickness
LVEF: Left ventricular ejection fraction
LVIDd: Left ventricular internal diameter
LVMI: Left ventricular mass index
LVPWd: Left ventricular posterior wall
