# Supplementary material for: Cardiac function assessed by myocardial deformation in adult polycystic kidney disease patients
Source: BMC Nephrol. 2019 Aug 16;20:324. doi: 10.1186/s12882-019-1500-1 (PMC6697983; doi:10.1186/s12882-019-1500-1)
Supplement: Supplementary file 1 — Table S1A. Associations with GLS, Linear Regression (those who were on Beta blocker were excluded from analysis). Table S1B. Associations with E/SRe, Linear Regression (those who were on Beta blocker were excluded from analysis). Table S2A. Associations with GLS: Logistic regression (those who were on Beta blocker were excluded from analysis). Table S2B. Associations with E/SRe: Logistic regression (those who were on Beta blocker were excluded from analysis). (DOCX 18 kb) [file 12882_2019_1500_MOESM1_ESM.docx]

Additional file 1: Table S1A Associations with GLS, Linear Regression (those who were on Beta blocker were excluded from analysis)

| Variable | Univariate | | Multiple linear regression with backward model selection | |
| --- | --- | --- | --- | --- |
|  | Beta coefficient (95% CI) | P value | Beta coefficient (95% CI) | P value |
| Age | 0.002 (-0.04,0.04) | 0.90 | 0.053 (0.002,0.11) | 0.04 |
| Gender | 1.56 (0.65,2.48) | 0.001 | 1.78 (0.80,2.76) | <0.001 |
| Diastolic Blood Pressure, mmHg | 0.053 (0.007,0.010) | 0.023 | 0.061 (0.012,0.11) | 0.015 |
| Estimated GFR, per 10 mL/min/1.73m^2^ | 0.030 (-0.12,0.18) | 0.70 | 0.22 (0.02,0.42) | 0.03 |
| Ht-Indexed Total Kidney Vol, per 100 cm^3^/m | 0.004 (-0.06,0.068) | 0.91 |  |  |
| BMI, kg/m^2^ | 0.039 (-0.06,0.13) | 0.42 |  |  |
| Fasting Glucose, mg/dL | 0.03 (-0.02,0.08) | 0.25 |  |  |
| HDL cholesterol, mg/dL | -0.034 (-0.07,0) | 0.05 |  |  |
| Triglycerides, mg/dL | 0.006 (-0.001,0.01) | 0.10 |  |  |
| Hemoglobin, g/dL | 0.39 (0.04,0.74) | 0.03 |  |  |

Additional file 1: Table S1B Associations with E/SRe, Linear Regression (those who were on Beta blocker were excluded from analysis)

| Variable | Univariate | | Multiple linear regression with backward model selection | |
| --- | --- | --- | --- | --- |
|  | Beta coefficient (95% CI) | P value | Beta coefficient (95% CI) | P value |
| Age | 0.21 (-0.26, 0.69) | 0.38 |  |  |
| Systolic Blood Pressure, mmHg | 0.44 (0.01, 0.86) | 0.04 | 0.43 (0.01, 0.85) | 0.047 |
| Estimated GFR, per 10 mL/min/1.73m^2^ | 0.60 (-1.3, 2.5) | 0.54 |  |  |
| BMI, kg/m2 | 0.69 (-0.52,1.90) | 0.26 |  |  |
| Microalbumin/creatinine ratio, mg/g | 0.02 (-0.04, 0.08) | 0.53 |  |  |
| ACE Inhibitor | -13.0 (-26.5, 0.46) | 0.06 | -14.1 (-27.7,-0.5) | 0.042 |

Additional file 1: Table S2A Associations with GLS: Logistic regression (those who were on Beta blocker were excluded from analysis)

| Variable | Univariate | | Multiple logistic regression with backward model selection | |
| --- | --- | --- | --- | --- |
|  | Odds ratio (95% CI) | P value | Odds ratio (95% CI) | P value |
| Age | 1.0 (0.97, 1.04) | 0.84 |  |  |
| Gender | 4.6 (1.9, 11.1) | <0.001 | 5.1 (2.0, 12.8) | <0.001 |
| Diastolic Blood Pressure, mmHg | 1.03 (0.98, 1.07) | 0.16 |  |  |
| Estimated GFR, per 10 mL/min/1.73m^2^ | 0.97 (0.85, 1.10) | 0.65 |  |  |
| BMI, kg/m^2^ | 1.03 (0.95, 1.12) | 0.44 |  |  |
| Fasting Glucose, mg/dL | 1.05 (1.0, 1.10) | 0.04 |  |  |
| HDL cholesterol, mg/dL | 0.98 (0.95, 1.01) | 0.11 |  |  |
| Triglycerides, mg/dL | 1.01 (0.99, 1.02) | 0.11 |  |  |
| Hemoglobin, g/dL | 1.52 (1.09, 2.13) | 0.01 |  |  |
| Ht-Indexed Total Kidney Vol, per 100 cm^3^/m | 1.06 (0.99, 1.12) | 0.06 |  |  |

Additional file 1: Table S2B Associations with E/SRe: Logistic regression (those who were on Beta blocker were excluded from analysis)

| Variable | Univariate | | Multiple logistic regression with backward model selection | |
| --- | --- | --- | --- | --- |
|  | Odds ratio (95% CI) | P value | Odds ratio (95% CI) | P value |
| Age | 1.01 (0.98, 1.05) | 0.42 |  |  |
| Systolic Blood Pressure, mmHg | 1.03 (0.99, 1.06) | 0.06 |  |  |
| Estimated GFR, per 10 mL/min/1.73m^2^ | 0.96 (0.85, 1.10) | 0.57 |  |  |
| BMI, kg/m2 | 1.12 (1.02, 1.23) | 0.02 | 1.12 (1.02, 1.23) | 0.02 |
| Microalbumin/creatinine ratio, mg/g | 1.0 (0.99, 1.01) | 0.19 |  |  |
| ACE Inhibitor | 0.68 (0.28, 1.71) | 0.41 |  |  |
